# Autosomal and X-Linked Additive Genetic Variation for Lifespan and Aging: Comparisons Within and Between the Sexes in *Drosophila melanogaster*

**DOI:** 10.1534/g3.116.028308

**Published:** 2016-09-27

**Authors:** Robert M. Griffin, Holger Schielzeth, Urban Friberg

**Affiliations:** * Department of Evolutionary Biology, Uppsala University, 752 36, Sweden; † Department of Biology, University of Turku, 20014, Finland; ‡ Department of Evolutionary Biology, Bielefeld University, 33615, Germany; § Department of Population Ecology, Institute of Ecology, Friedrich Schiller University Jena, 07743, Germany; ** IFM Biology, AVIAN Behavioural Genomics and Physiology Group, Linköping University, 581 83, Sweden

**Keywords:** dosage compensation, faster X, intersexual genetic correlation, sexual dimorphism, X chromosome

## Abstract

Theory makes several predictions concerning differences in genetic variation between the X chromosome and the autosomes due to male X hemizygosity. The X chromosome should: (i) typically show relatively less standing genetic variation than the autosomes, (ii) exhibit more variation in males compared to females because of dosage compensation, and (iii) potentially be enriched with sex-specific genetic variation. Here, we address each of these predictions for lifespan and aging in *Drosophila melanogaster*. To achieve unbiased estimates of X and autosomal additive genetic variance, we use 80 chromosome substitution lines; 40 for the X chromosome and 40 combining the two major autosomes, which we assay for sex-specific and cross-sex genetic (co)variation. We find significant X and autosomal additive genetic variance for both traits in both sexes (with reservation for X-linked variation of aging in females), but no conclusive evidence for depletion of X-linked variation (measured through females). Males display more X-linked variation for lifespan than females, but it is unclear if this is due to dosage compensation since also autosomal variation is larger in males. Finally, our results suggest that the X chromosome is enriched for sex-specific genetic variation in lifespan but results were less conclusive for aging overall. Collectively, these results suggest that the X chromosome has reduced capacity to respond to sexually concordant selection on lifespan from standing genetic variation, while its ability to respond to sexually antagonistic selection may be augmented.

The X chromosome is present as only a single copy in males. Together with an unusual inheritance pattern, this presumably exposes the X chromosome to population genetic parameter values that differ from those of the autosomes (Vicoso and Charlesworth 2006; Ellegren 2009). As a result, both the amount and the type of molecular variation potentially differ between the X chromosome and the autosomes. The direction of this difference depends on a range of factors (Ellegren 2009), and it is not obvious to what extent differences observed at the molecular level translate to phenotypic variation (Dean and Mank 2014).

Theory typically predicts that, within populations, the X chromosome should be depleted of molecular genetic variation relative to the autosomes. This prediction follows from the hemizygosity of the X chromosome in males, which both reduces the effective population size of the X to three-quarters that of the autosomes and results in more efficient selection on X-linked mutations (Avery 1984; Charlesworth *et al.* 1987). However, because the X chromosome spends two-thirds of its time in females, there are many other factors with the potential to alter the relative amount of genetic variation at the X chromosome (reviewed in Ellegren 2009).

Empirical investigations into the relative molecular variation at the X chromosome and the autosomes of *Drosophila melanogaster* have shown mixed results. In general, the X chromosome hosts less molecular variation in non-African populations (Hutter *et al.* 2007; Mackay *et al.* 2012), while this effect is less pronounced, and commonly reversed, in ancestral African populations (Hutter *et al.* 2007). With respect to nonsynonymous sites, which are presumably subject to selection, lower molecular variation on the X chromosome is, however, consistently reported in all populations studied so far (Langley *et al.* 2012; Campos *et al.* 2013). Relatively lower molecular variation at the X chromosome has also been reported for humans (*e.g.*, Arbiza *et al.* 2014) and the Z chromosome in birds (*e.g.*, Hogner *et al.* 2012).

It seems reasonable to expect that reduced X-linked variation at functional molecular sites should also reduce X-linked genetic variation for phenotypic traits. GWAS and QTL analyses of humans (Yang *et al.* 2011; Tukiainen *et al.* 2014) and a bird species (Robinson *et al.* 2013; Santure *et al.* 2013) indeed support this prediction, but investigations of *D. melanogaster* to date do not. Across two studies, involving a total of 28 morphological traits, the average proportion of the total genetic variation assigned to the X chromosome was estimated to be 19.6% (Cowley *et al.* 1986; Cowley and Atchley 1988). Although there was variation between traits, the average is not less than what would be predicted from the relative size of the X chromosome [15.6 and 18.8%, based on the proportion of protein coding genes and euchromatin respectively (*D. melanogaster* genome release 5.30)]. Studies of fitness (Gibson *et al.* 2002) and locomotory activity (Long and Rice 2007) suggest that the contribution of the X chromosome to genetic variation could be disproportionately large in this species.

A factor that may complicate the link between genetic variation at the molecular and the phenotypic trait level, specific to differences between the autosomes and the X chromosome, is dosage compensation. When complete, dosage compensation should normally result in elevated X-linked standing genetic variation in males compared to females, because the male population effectively consists of only homozygous individuals for X-linked loci (Reinhold and Engqvist 2013). However, dosage compensation may also increase X-linked genetic variation in females if selection for higher gene expression in males increases expression in females as a correlated response (Prince *et al.* 2010; Xiong *et al.* 2010; Mank *et al.* 2011; Wright and Mank 2012; Allen *et al.* 2013).

Two other factors that may also complicate the link between genetic variation at the molecular and phenotypic level, in a comparison between the X chromosome and the autosomes, are sexually antagonistic allelic variants and regulatory elements with sex-specific effects. Since sexually antagonistic variants are exposed to opposing selection in males and females, net selection will in general be weaker on such variants compared to mutations selected concordantly in both sexes. Therefore, they may maintain more variation than concordantly selected variants, also when they are not maintained at a balanced polymorphism (Connallon and Clark 2012). Earlier theory suggested that sexually antagonistic variation should be shifted toward the X chromosome (Rice 1984), while more recent theory has suggested the opposite (Fry 2010; Connallon and Clark 2010).

Sex-specific regulators, which evolve to resolve sexual conflict over gene expression, are also expected to host elevated levels of variation at mutation-selection equilibrium, as they are primarily exposed to selection in only one sex (Morrow and Connallon 2013). If such regulatory elements are positioned predominantly *in cis* to the genes they influence, these may also have a skewed chromosomal distribution. Much of the sex-specific and sexually antagonistic variation is probably hosted in noncoding regions with regulatory effects, where they have a very small influence on molecular variation in general, while they may have sizable effects on variation at the phenotypic level. A prediction following uneven chromosomal distribution of sex-specific regulators is that the intersexual genetic correlation (*r*_MF_) should differ between chromosome types.

In this study, we use autosome and X chromosome substitution lines to study autosomal and X-linked additive genetic (co)variation within and between the sexes, for lifespan and aging in *D. melanogaster*. By randomly sampling chromosomal copies from one large outbred laboratory population we attain unbiased estimates of additive genetic variation. Using this method, we address the following three questions: (i) does the X chromosome show reduced levels of additive genetic variation, (ii) does the X chromosome maintain more additive genetic variation in males compared to females, and (iii) does the X chromosome harbor relatively more sex-specific additive genetic variation than the autosomes? By assessing the genomic distribution of variation in lifespan and aging, this study expands on a previous study of the same population, which reported substantial sex-specific genetic variation for both of these traits when genetic variation was estimated for the whole genome as a single unit (Lehtovaara *et al.* 2013).

## Materials and Methods

### Experimental population

In our experiment, we used a laboratory adapted population of *D. melanogaster* (Dahomey), originating from a sample of wild-caught flies collected in Benin (Africa) over 40 years ago. Dahomey has since been kept as a large outbred population, with overlapping generations and in constant conditions (12:12 light-dark cycle, 60% humidity, 25°, and on a standard yeast-sugar diet). All flies in this experiment were kept under these standard conditions throughout.

### Construction of X and autosome substitution lines

**Figure 1:**
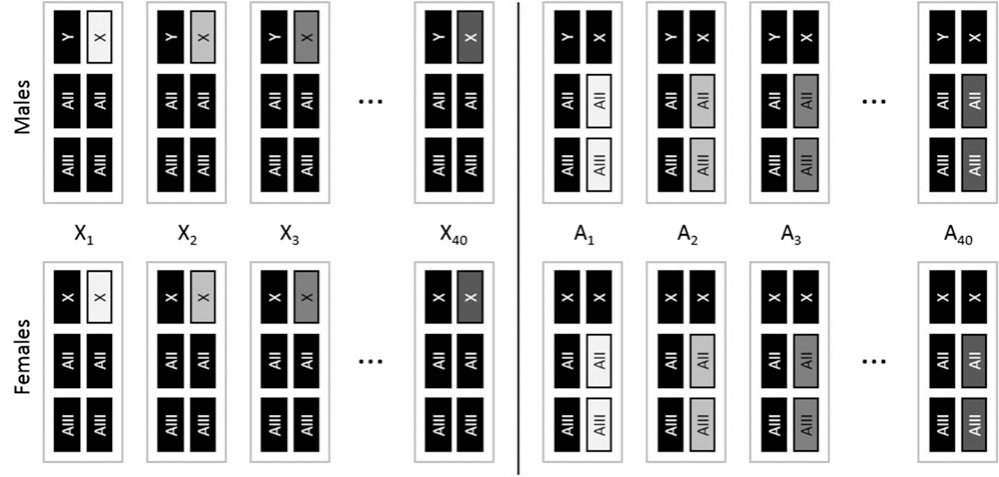
Schematic of X-lines and A-lines. Gray rectangles depict cloned chromosome(s) within a line, while black rectangles depict chromosomes that vary randomly between individuals within lines. Sex chromosomes are symbolized with X and Y and the major autosomes are symbolized with AII and AIII. The fourth dot chromosome (< 1% of the genome) was not controlled in the experiment and is omitted from the figure. See Figure S1 for a detailed schematic of the line construction process.

The genome of *D. melanogaster* is composed of the sex chromosomes (X and Y), two major autosomes (AII and AIII), and the small fourth dot chromosome (AIV, < 1% of the genome). To study the autosomal contribution to additive genetic variance for lifespan and aging, we randomly sampled 40 copies of chromosomes AII and AIII, and clonally amplified them as haploid pairs into random genetic backgrounds. Within each autosome substitution line (A-line), all individuals share an identical copy of AII and AIII, while all other chromosome copies vary randomly among individuals (Figure 1). To study the contribution of the X chromosome to additive genetic variance for lifespan and aging, we randomly sampled 40 copies of the X chromosome and clonally amplified them into random genetic backgrounds. Within each X chromosome substitution line (X-line), all individuals share one identical X chromosome and vary randomly with respect to all other chromosomes (Figure 1). Because the genotypic value for each randomly sampled X chromosome, and each randomly sampled pair of autosomes AII and AIII, was measured in a large number of random genetic backgrounds in each sex, variation among lines can be used to calculate the additive genetic variance separately for each sex and chromosome type. These estimates are devoid of dominance variation, but could include a minor component of variation caused by epistatic interactions, within and between cloned chromosome copies (see Friberg *et al.* 2005; Rice *et al.* 2005; Lehtovaara *et al.* 2013 for discussion).

A-lines and X-lines were constructed by first taking 80 randomly selected Dahomey males and crossing them individually to virgin DXCG females (C[1]DX, *y*, *f*/Y; T[2;3] *bwD*, *in*, *p^p^*, *rdgC*, *ri*, *st*/T[2;3] *bwD*, *in*, *p^p^*, *rdgC*, *ri*, *st*) (see Supplemental Material, Figure S1 throughout). Sons from these crosses inherited their father’s wild-type copy of the X chromosome and a copy each of his wild-type autosomes. From their mother, they inherited a Y chromosome and a phenotypically marked translocation between the major autosomes, which forces the homologous AII and AIII chromosome copies to cosegregate. To construct the A-lines, we took one male offspring from each of the 40 above crosses and mated them individually to virgin CG-D females from a population homozygous for the aforementioned autosomal translocation, but with genetically variable wild-type Dahomey X chromosomes. This procedure replaced the one X chromosome associated with the founder male with randomly sampled Dahomey X chromosomes, and cloned the focal sets of AII and AIII chromosomes. Each A-line was maintained at a size of 40 males, mated to 80 CG-D females for three generations prior to producing focal flies.

To construct the X-lines, we took multiple sons from each of the remaining 40 initial crosses (all sons from each cross carry the same X chromosome copy) and mated them to virgin DX-D females, a population where females carry the aforementioned compound X chromosome (C[1]DX,*y*,*f*)/Y) placed with genetically variable wild-type Dahomey autosomes, to remove the autosomes associated with the founder male. Sons from each of these crosses were then mated to virgin DX-D females to remove the autosomal translocation. This procedure replaced autosomes associated with the founder male with randomly sampled Dahomey autosomes. Each X-line was maintained at a size of 40 males mated to 80 DX-D females for three generations before focal flies were produced. The crossing scheme to produce and maintain the X- and A-lines are described in detail in the Supplemental Material (Figure S1).

### Lifespan and aging assay

Lifespan and aging were both estimated from 200 focal flies of each sex and line, split equally among four replicate vials, totaling 32,000 focal flies. For each replicate, focal flies were produced by either crossing 45 males from each X- and A-line to (i) 90 virgin Dahomey females across three vials to produce focal females, or (ii) to 90 virgin DX-D females across three vials to produce focal males. Once the parental flies were transferred from the oviposition vials, the number of eggs was manipulated to standardize the number of viable larvae to 150 per vial.

Ten days after egg laying, virgin focal flies were collected under light CO_2_ anesthesia (< 4 min of exposure) into vials of 56 individuals per sex and line. These were paired with 56 opposite sexed flies homozygous for a recessive dark body pigment mutation (*ebony*, earlier introgressed into the Dahomey background) and allowed to interact and mate for 72 hr. Ebony flies were subsequently removed and discarded and 50 focal flies were randomly selected (after removing any dead flies) and transferred to a fresh vial under light CO_2_ anesthesia. After 24 hr, the flies were transferred to fresh food without anesthesia. Every 48 hr, from this point onwards, we transferred the focal flies to fresh vials without anesthesia, scored mortality, and discarded dead flies.

### Outlier vial removal

Visual examination of the mean female lifespan per vial revealed a bimodal distribution, with a small group of vials hosting unusually short-lived females, suggesting that a strong extrinsic factor (*e.g.*, disease) affected survival in these vials. Due to the nature of the distribution, vials presumably affected were easily separated out, having an average lifespan of < 51 d (Figure S2). Since we were interested in genetic variation between lines, we tested if there was a genetic component to the low lifespan vials. To do this, we first removed the low scoring vials and then tested whether female lifespan of lines not having a low scoring vial was larger than lines which had a low scoring vial. No difference between these groups of lines was detected [mean difference (lines without low scoring vial – lines with low scoring vial) (days): X-lines = 0.14, *t*_38_ = 0.22, *P* = 0.41; A-lines = 0.36, *t*_38_ = 0.25, *P* = 0.40; all lines = −0.19, *t*_78_ = −0.23, *P* = 0.59, all *P*-values one-tailed). Hence, there was no indication that lines with outlier vials were more short-lived than other lines due to their genotype. Visual inspection of the distribution of 400 female and 400 male vials from a previous study (Lehtovaara *et al.* 2013), where the same population was studied under similar experimental conditions, showed no excess of low scoring vials. Taken together, this suggests that the small group of low scoring vials represent true outliers. Therefore, we present results from analyses excluding these vial. However, results including all vials are reported in Table S1.

### Bayesian lifespan models

Lifespan data were analyzed separately for the two line types (X- and A-lines), using mixed-effects models fitted by Markov chain Monte Carlo (MCMC) sampling as implemented in the MCMCglmm package (Hadfield 2010) in R 3.1.2 (R Core Team 2014). Lifespan data were modeled assuming Gaussian error distributions with lifespan in each sex treated as separate response variables. This multi-response model approach allowed us to efficiently estimate intersexual genetic correlations. Line and vial were fitted as random effects and sex-specific fixed effects were fitted to account for the four batches of replicates. Fixed effect dummy variables were centered, such that the intercept estimates the global mean rather than the average lifespans for one of the batches (Schielzeth 2010). The vial random effect captures environmental variation associated with each vial, but also genotype-by-batch interactions, since there was a single vial per line and batch. Unstructured variance-covariance matrices were formed, each containing variance-covariance estimates for both sexes, with one 2 × 2 matrix for the A-lines and one 2 × 2 matrix for the X-lines. Vial and residual variance-covariance matrices had off-diagonal elements constrained to zero, because each vial and fly can only represent one sex and line type and therefore has the covariance structure undefined. The final model in R code was MCMCglmm(cbind(LSf,LSm) ∼ trait − 1 + trait:batch2 + trait:batch3 + trait:batch4, random = ∼us(trait):Line + idh(trait):Vial, rcov = ∼idh(trait):units, family = rep(“gaussian”, 2)), where LSf and LSm are individual lifespans of females and males, respectively, and batch2, batch3, and batch4 are the dummy-coded and centered identifiers for batches 2–4, respectively.

We used parameter-expanded priors with a belief (shape) parameter *ν* = 2 for the variance-covariance matrices of the random effects and inverse-Wishart priors with *ν* = 0.002 for residual variances (recommended in the documentation of the MCMCglmm package, Hadfield 2010). A sensitivity analysis regarding different choices of the degree of the belief (shape) parameter *ν* for the random effects showed robustness between *ν* = 0.002 and *ν* = 3. Four independent MCMC chains, two for each line type, were run for 1,100,000 iterations, with a burn-in of 100,000 iterations and a thinning interval of 1000 iterations. Convergence was checked visually and, using the Gelman-Rubin criterion, applied to two independent chains for each line type (all upper 95% confidence limit of potential scale inflation factors ≤ 1.05).

### Bayesian aging models

Gompertz mortality functions of the form µ(*t*) = α*e*^β^*^t^* (where µ(*t*) is the rate of mortality at age *t*) allow decomposition of lifespan into components α, the initial mortality, and the rate of aging β. We estimate these two parameters at the level of the vial, using the program WinModest (Pletcher 1999). Four estimates of each parameter, one per batch, were made for each of the 160 combinations of line and sex. The two parameters α and β were strongly negatively correlated (*r* = −0.94, 95% CI −0.93–0.95, *P* < 10^−15^, with α log transformed to account for the highly skewed distribution). Therefore, we decided to model only the aging parameter β in multi-response models, similar to the lifespan models described above, but without the random effect of vial as there was only one estimate of the population parameter β per vial. We also implemented a bivariate, nonlinear mixed model in OpenBUGS 3.2.3 (Lunn *et al.* 2009) with parameter β allowed to vary and covary between lines and sexes, but the model did not converge for the critical parameter of the genetic correlations. Hence, we present the results of the two-step analysis here (using WinModest estimates of β as data in the MCMCglmm model as described above).

### Summaries of model fits for variances and covariances

With the above models, we estimated the line variance (*V*_L_), the vial variance (*V*_V_), and the residual variance (*V*_R_) separately for the two sexes, and the line covariance among sexes (*Cov*_MF_). The total phenotypic variance (*V*_P_) was reconstructed as the sum *V*_P_ = *V*_L_ + *V*_V_ + *V*_R_, again separately for the two sexes. Since lines were cloned for haploid chromosomes, additive genetic variance was calculated by multiplying the line variances by 2, with the exception of the male X-lines (because the X is hemizygous in males). The line covariance was converted to an intersexual additive genetic correlation by *r*_MF_ = *Cov*_MF_/(√*V*_LF_ * √*V*_LM_). One of the key advantages of the MCMC sampling approach is that we can form sums, ratios, and differences of (co)variances for the entire chain, and thus get samples from the posterior distribution of these quantities. For estimating differences between independent runs for X and A lines, we linked the chains in random order and calculated the differences between the (randomly selected) samples from the posterior distribution to get the distribution of differences. We summarize posterior distributions by their mean, and SD as the Bayesian SE and 95% interquantile range (95% CI, *i.e.*, credible interval). However, male to female ratios of X-linked genetic variation showed significant positive outliers due to low genetic variance in females (including some samples from the posterior distribution close to zero) leading to excessively high ratios. These highly skewed distributions are poorly summarized by the mean and the SD, and we present the median and the interquartile range of the posterior distribution instead.

### REML fits and likelihood ratio tests

In addition to the Bayesian analysis, we fitted models by restricted maximum likelihood (REML) in ASReml 4.1 (Gilmour *et al.* 2009) to the same data. Model estimates of these REML-fitted models for lifespan were very similar to the Bayesian model fits and confirmed the robustness to Bayesian estimation. Multivariate models for aging, however, did not converge for the intersexual covariance of X chromosomal lines and we were therefore not able to directly compare ASReml fits with MCMCglmm fits for aging. An advantage of the REML framework is that we can constrain parameters of interest to the values predicted under the null hypothesis (null model) and test an alternative model in which the parameters of interest are unconstrained. For the full model, we treated each sex × line type combination as a separate trait and thus fitted a four-trait model jointly for both line types. The model included a fixed effect for each batch for each trait, as well as a vial and a line random effect component. For the vial random effect, we estimated the four variances, while covariances were undefined by the data and hence constrained to zero in the model. For the random effect of line, we estimated the four variances for the four sex × line type combination as well as the covariances between sexes within line types. The four potential covariances across line types were undefined (because any particular hemiclone was either of the autosomal or the X chromosome type) and were hence constrained to zero in the model. We derived *P*-values for three specific null hypotheses using likelihood ratio tests (LRT): (i) [H0] the ratio of the X:A chromosome standing genetic variance is directly proportional to the DNA content by constraining the variance ratio to be of the predicted values (all constraints according to the instructions in the manual, Gilmour *et al.* 2009, chapter 7.9); (ii) [H0] the ratio of variance of additive genetic variances is equal in males and females by constraining the X-line variances to be equal in males relative to females, and (iii) [H0] the cross-sex genetic correlations are equal for the X chromosome and the autosomes by constraining the correlations to be equal between chromosome types. Furthermore, we fitted univariate models for each sex × line type combination to test for the statistical significance of the line variance using LRT. These models were fitted using the lme4 package (Bates *et al.* 2015) and converged for all lifespan and aging traits. The alternative of testing individual line variance in the multivariate models fitted in ASReml yielded almost identical results for lifespan, while LRT were not possible in multivariate models of aging (see above).

### Data availability

Data are available from the Dryad Digital Repository (http://dx.doi.org/10.5061/dryad.11q0d).

## Results

**Figure 2:**
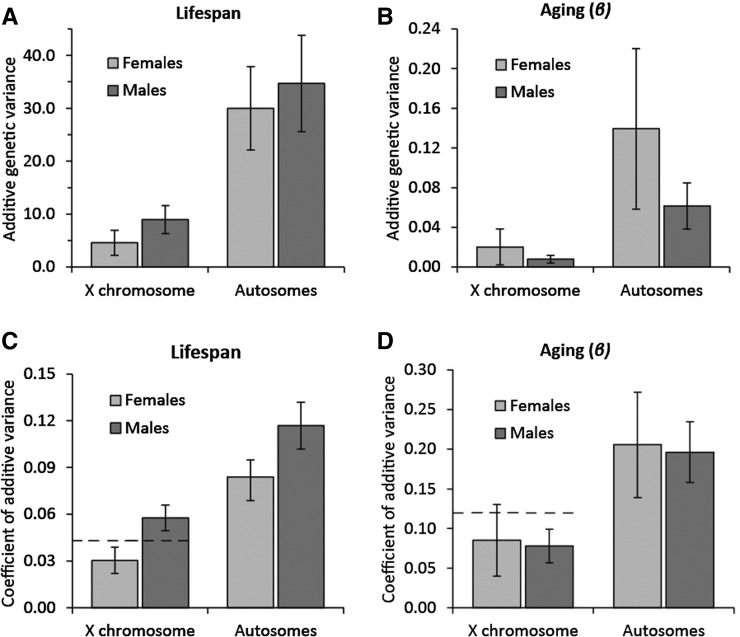
Additive genetic variance in lifespan and aging across chromosome types. Additive genetic variation in (A) lifespan and (B) aging (β × 100) for the X chromosome and the autosomes. The coefficient of additive genetic variation (CV_A_) for lifespan (C) and aging (D). Dashed lines indicate twice the X-linked female additive genetic variation on this scale. Error bars depict SE.

Estimates of X chromosome and autosomal line variance for lifespan, and hence also the corresponding estimates of additive genetic variance (see *Materials and Methods*), were significantly different from zero for both sexes (LRT on REML-fitted models: *X*^2^_1_ = 9.26, *P* = 0.002 for female *X*-lines, *X*^2^_1_ > 70.0, *P* < 0.001 for all others; Figure 2, Table 1, and Table S2). Chromosomal variances for aging were, in general, estimated with more uncertainty, but were significantly larger than zero for male and female A lines (LRT on REML-fitted models: both *X*^2^_1_ > 14.0, *P* < 0.001), male *X*-lines (*X*^2^_1_ = 6.40, *P* = 0.011), and marginally nonsignificant for female *X*-lines (*X*^2^_1_ = 3.83, *P* = 0.050) (Figure 2, Figure S3, and Table 1).

**Table 1:** Mean and variance estimates for lifespan and aging

		Autosomes		X-Chromosomes	
		Female	Male	Female	Male
Lifespan	Mean	64.75 ± 0.63	50.03 ± 0.69	67.80 ± 0.34	51.36 ± 0.50
	CI	63.45–65.92	48.63–51.31	67.13–68.48	50.38–52.33
	*V*_L_	15.00 ± 3.94	17.36 ± 4.56	2.28 ± 1.18	8.95 ± 2.64
	CI	8.85–24.17	10.42–27.93	0.28–4.96	5.08–15.38
	*V*_V_	4.78 ± 0.88	4.82 ± 0.91	5.96 ± 1.13	3.19 ± 0.69
	CI	3.24–6.68	3.28–6.78	4.14–8.62	2.00–4.68
	*V*_R_	73.72 ± 1.24	89.92 ± 1.43	60.95 ± 1.04	98.84 ± 1.59
	CI	71.38–76.14	87.19–92.70	58.92–62.98	95.76–102.02
	*V*_P_	93.49 ± 4.14	112.10 ± 4.75	69.19 ± 1.68	110.98 ± 3.11
	CI	86.74–103.27	104.29–122.6	66.03–72.64	105.91–117.96
	*V*_A_	30.00 ± 7.88	34.71 ± 9.12	4.57 ± 2.36	8.95 ± 2.64
	CI	17.70–48.35	20.84–55.87	0.57–9.93	5.08–15.38
	*CV*_A_	0.08 ± 0.01	0.12 ± 0.02	0.03 ± 0.01	0.06 ± 0.01
	CI	0.06–0.11	0.09–0.15	0.01–0.05	0.04–0.08
Aging (β)	Mean	17.29 ± 0.59	12.41 ± 0.34	14.77 ± 0.34	10.95 ± 0.20
	CI	16.16–18.44	11.76–13.11	14.10–15.47	10.56–11.33
	*V*_L_	6.97 ± 4.05	3.07 ± 1.16	1.01 ± 0.91	0.78 ± 0.40
	CI	0.43–15.38	1.21–5.72	0.00–3.28	0.16–1.72
	*V*_R_	25.86 ± 3.93	6.66 ± 0.87	12.55 ± 1.67	3.03 ± 0.41
	CI	19.33–34.49	5.16–8.65	9.60–16.07	2.32–3.93
	*V*_P_	32.83 ± 4.50	9.73 ± 1.27	13.56 ± 1.70	3.81 ± 0.48
	CI	25.44–42.28	7.60–12.51	10.65–17.22	2.98–4.89
	*V*_A_	13.94 ± 8.10	6.14 ± 2.32	2.02 ± 1.82	0.78 ± 0.40
	CI	0.86–30.77	2.41–11.43	0.01–6.56	0.16–1.72
	*CV*_A_	0.206 ± 0.066	0.196 ± 0.038	0.085 ± 0.045	0.078 ± 0.021
	CI	0.053–0.326	0.125–0.274	0.006–0.172	0.036–0.119

Values provided are estimates from the MCMCglmm model followed by the SEM, and 95% credible intervals (CI). Mean lifespan is given in days, and mean aging is given for values of β (× 100), with estimates of line (*V*_L_), vial (*V*_V_) (could only be estimated for lifespan), residual (*V*_R_), and phenotypic (*V*_P_) variance. Additive genetic variance (*V*_A_) and the coefficient of additive genetic (*CV*_A_) were derived from line variance and mean estimates.

### Comparing X to autosomal additive genetic variance

To evaluate if the contribution of the X chromosome to additive genetic variance is different to that expected from its size, we focus on females because the relative contribution of the X is complicated by dosage compensation in males. Since size, composition, and gene content potentially varies between chromosomes, it is not obvious what constitutes the best unit for calculating the proportion of the active genome which is X-linked, but two metrics that should provide good approximations are the proportion of euchromatin and the proportion of genes situated on the X chromosome. In *D. melanogaster*, the X chromosome hosts 18.8% of the euchromatin and 15.6% of the genes (*D. melanogaster* genome Release 5.30). Point estimates suggest a moderate to slight depletion of X-linked additive genetic variance, although this was far from significant (female X-linkage of lifespan *V*_A_ = 13.5% ± 6.7%, 95% CI = 1.9–28.5%; female X-linkage of aging *V*_A_ = 15.7% ± 17.0%, 95% CI = 0.1–67.1%), a result also confirmed by likelihood ratio testing on REML-fitted models for lifespan (*X*^2^_1_ = 0.61, *P* = 0.43 for a ratio-constraint based on euchromatin, *X*^2^_1_ = 0.086, *P* = 0.79 for a ratio-constraint based on gene content).

### Comparing X-linked additive genetic variance in males and females

To test if X chromosome hemizygosity and associated dosage compensation cause males to have more X-linked additive genetic variance than females, we first compared X-linked *V*_A_ in males (*V*_AMX_) and females (*V*_AFX_) (where subscript F and M denote female or male respectively, and subscript X denotes the X chromosome). The ratio of male to female X-linked *V*_A_ (*V*_AMX_/*V*_AFX_) was estimated to be larger, but not significantly different from, 1 for lifespan (median = 2.05, interquartile range: 1.40–3.09, 95% CI = 0.75–12.82), and was estimated to be lower, but not significantly different from 1 for aging (median 0.47, interquartile range: 0.22–1.26, 95% CI = 0.07–74.73). Likelihood ratio tests suggest a ratio significantly > 1 for lifespan (*X*^2^_1_ = 7.79, *P* = 0.0053). These comparisons do however not take into account that this population displays sexual dimorphism for lifespan and aging (Table 1) and, since variance is expected to scale with the mean, this has to be taken into account. The coefficient of additive variation (CV_A_) provides a mean-standardized scale-free measure of variation and therefore provides more suitable estimates for comparison. The ratio of the male to female CV_A_ for the X chromosome is significantly > 1 for lifespan (median = 1.88, interquartile range: 1.56–2.32, 95% CI = 1.14–4.70), and again not significantly different from 1 for aging (median: 0.93, interquartile range: 0.63–1.51, 95% CI = 0.35–11.67). Any differences in the genetic variance in males compared to females may, however, not be restricted to the X chromosome, as a trend for a male to female ratio of *V*_A_ (*V*_AMA_/*V*_AFA_) above one for lifespan was also observed for the autosomes (median = 1.15, interquartile range: 0.94–1.52, 95% CI = 0.63–2.12, LRT: *X*^2^_1_ = 0.22, *P* = 0.64), as well as a ratio below one for aging (median = 0.46, interquartile range: 0.28–0.78, 95% CI = 0.13–7.16). Using CVs to correct for sex differences in means shows significantly more autosomal variation in males for lifespan (median = 1.39, interquartile range: 1.26–1.54, 95% CI = 1.02–1.88) and no difference for aging (median: 0.94, interquartile range: 0.74–1.23, 95% CI = 0.49–3.83). To take the autosomes into account when evaluating if males have comparatively more X-linked *V*_A_ than females, we calculated (CV_AMX_/CV_AFX_)/(CV_AMA_/CV_AFA_). This ratio is not different from 1 for lifespan (median = 1.36, interquartile range: 1.10–1.72, 95% CI = 0.75–3.60), and not for aging either (median: 0.98, interquartile range: 0.60–1.71, 95% CI = 0.19–12.90).

### Comparing the r_MF_ between the X and the autosomes

**Figure 3:**
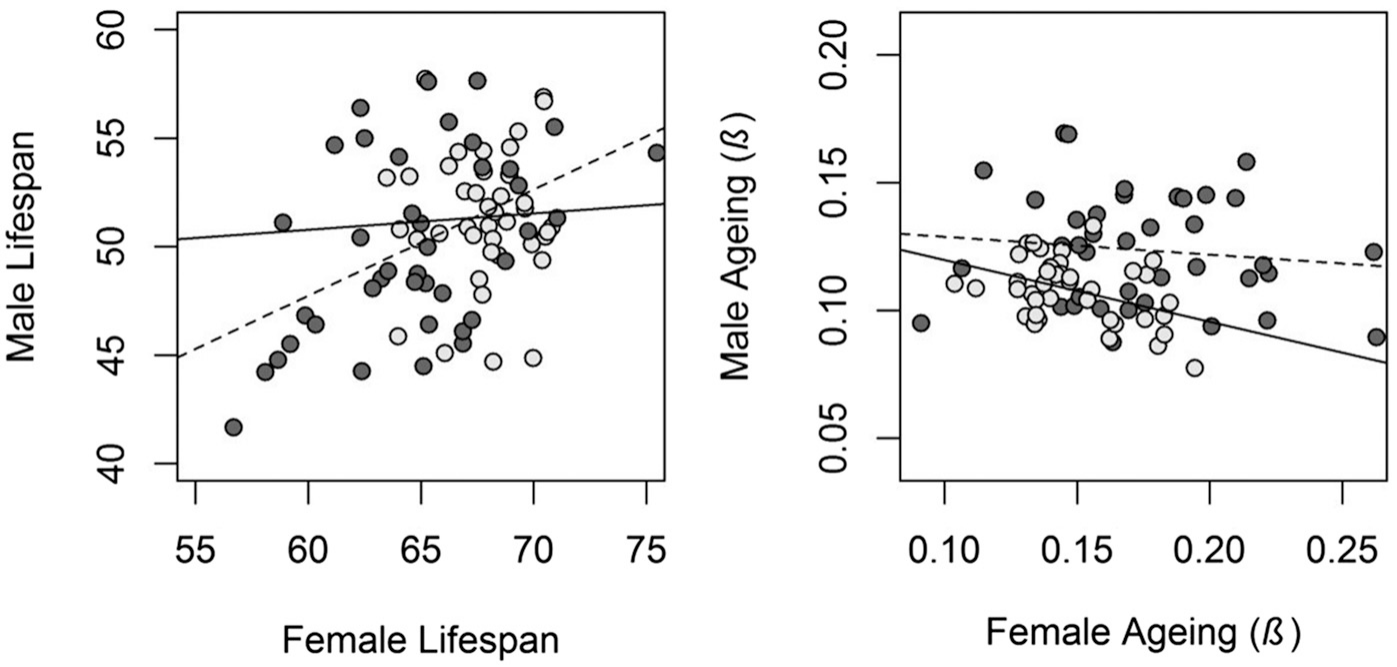
Scatterplot of male and female X- and A-line means for lifespan and aging. Light gray points and solid lines represent the X chromosome, and dark gray points and dashed lines represent the autosomes. The plot is scaled such that the steepness of the regression slopes reflects the strength of the correlation.

To test if the X chromosome is enriched for sex-specific additive genetic variance, we calculated and compared the intersexual additive genetic correlation (*r*_MF_) for the X chromosome and the autosomes. For lifespan, the *r*_MF_ of the autosomes was moderate and significantly greater than zero (*r*_MF-A_ = 0.50 ± 0.14, 95% CI = 0.19–0.74, Figure 3), while it was low and not significantly different from zero for the X chromosome (*r*_MF-X_ = 0.06 ± 0.24, 95% CI = −0.43 to 0.53, Figure 3). The *r*_MF_ for the autosomes was not statistically significantly different from the X chromosome, although the credible intervals only marginally overlap zero (lifespan *r*_MF-X_ – *r*_MF-A_ = −0.46 ± 0.28, 95% CI = −0.97 to 0.12, 111 of 2000 posterior samples, *i.e.*, 5.55%, were ≥ 0). Likelihood ratio testing showed a similar marginally nonsignificant result (LRT *X*^2^_1_ = 3.166, *P* = 0.075). The intersexual genetic correlation for aging was not significantly different from zero both for the autosomes (*r*_MF-A_ = −0.12 ± 0.28, 95% CI = −0.65 to 0.45, Figure 3) and the X chromosome (*r*_MF-X_ = −0.42 ± 0.38, 95% CI = −0.94 to 0.57, Figure 3), and these were not different from one another (*r*_MF-X_ − *r*_MF-A_ = −0.30 ± 0.48, 95% CI = −1.11 to 0.82, Figure 3).

## Discussion

In this study, we independently measured X chromosome and autosomal additive genetic variance in males and females for the life history traits lifespan and aging. Below, we compare these estimates and discuss them in the context of several hypotheses, which predict differences in the amount and type of genetic variation between the X and the autosomes, and between male and female X-linked variation. We also briefly discuss the implications of our results with respect to faster X evolution.

### Comparing X to autosomal additive genetic variance

Theory suggests that the X chromosome should be depleted of genetic variation (Haldane 1937; Avery 1984; Charlesworth *et al.* 1987). Point estimates of the traits studied here support this prediction, but relatively wide credible intervals preclude firm conclusion. These results point in the same direction as a study on the genomic distribution of *trans*-regulatory variation of gene expression in *D. melanogaster*, which found relatively lower levels of variation hosted on the X chromosome (Stocks *et al.* 2015).

Earlier studies of quantitative traits in *D. melanogaster* have, however, pointed to either no depletion (Cowley *et al.* 1986; Cowley and Atchley 1988) or even enrichment (Gibson *et al.* 2002; Long and Rice 2007) of X-linkage. The former two of these studies applied a statistical model based on several possibly invalid assumptions, which could potentially explain the lack of observed depletion. The latter studies used chromosome substitution lines and should, just as the present one, have produced largely unbiased estimates of additive genetic variation. These studies found large amounts of sexually antagonistic variation for fitness (Gibson *et al.* 2002) and locomotory activity (Long and Rice 2007). Some theories (Rice 1984), but not others (Fry 2010; Connallon and Clark 2010), suggest that sexually antagonistic variation should be shifted toward the X chromosome, and this could potentially explain the observed X-linked enrichment, rather than depletion, of variation for these traits.

Studies of quantitative traits in humans (Yang *et al.* 2011; Tukiainen *et al.* 2014) and birds (Robinson *et al.* 2013; Santure *et al.* 2013) suggest that the X and Z chromosomes are depleted of genetic variation. Depletion of X-linked variation for quantitative characters thus seems to be the general trend, while traits under strong sexually antagonistic selection may be exempt. Further studies on the relative X (Z) -linkage of quantitative traits, coupled with information on the direction of selection in each sex, and their *r*_MF_, are however required to test this hypothesis.

### Comparing X-linked additive genetic variance in males and females

X-linked genes are effectively homozygous in males when the X chromosome is fully dosage compensated. From this, it follows that X-linked variance should typically be higher in males than females (and two times higher when all variation is additive) (Reinhold and Engqvist 2013; Figure S4). This hypothesis has received mixed support from empirical studies comparing total male and female genetic variation across a broad range of species (Reinhold and Engqvist 2013; Wyman and Rowe 2014; Nakagawa *et al.* 2015). With respect to *D. melanogaster*, point estimates of a male bias in X-linked additive genetic variation have previously been found in 20 out of 22 morphological characters (Cowley *et al.* 1986; Cowley and Atchley 1988), as well as for locomotory activity (Long and Rice 2007) and fitness (Gibson *et al.* 2002).

Our results show significantly more X-linked variation in males than females for lifespan, but not for aging. For lifespan, the picture is complicated by the fact that autosomal variation is larger in males. Why males show more variation than females in general is not obvious, but could be related to deleterious mutations having a generally larger effect on fitness in males (Mallet *et al.* 2011; Sharp and Agrawal 2013), and thus generate more variation in this sex. If this effect carries over to traits closely connected to fitness, such as lifespan, this could potentially generate more variation for lifespan in males than females (*e.g.*, see Figure 2 in Kimber and Chippindale 2013). When also taking into account that autosomal variation is larger in males, we no longer see a significant excess of male X-linked variation. We do, however, note that the observed male to female ratio of X and autosomal CV ratios (1.36) is close to what is expected (1.41 = √2) when there is two times more male variation on a “square root scale.”

### Comparing the r_MF_ between the X and the autosomes

When the sexes have different phenotypic optima, which they yet have not reached, genetic variation becomes sexually antagonistic. The resolution to such intralocus sexual conflict is the evolution of sexual dimorphism through regulatory modifiers with sex-specific effects. Early theory suggested that the X chromosome should be enriched for sexually antagonistic variation (Rice 1984), something that later theory has questioned by suggesting that it could be reversed (Connallon and Clark 2010; Fry 2010). If modifiers develop *in cis*, a lower *r*_MF_ should be associated with the chromosomes which, at least in the past, have hosted more sexually antagonistic variation.

We estimate the *r*_MF_ for lifespan to be close to zero (0.06) for the X chromosome and moderate (0.50) for the autosomes. The difference between these estimates was marginally nonsignificant, but the fact that intermediate estimates (*r*_MF_ = 0.29 and *r*_MF_ = 0.43; values for two social environments) were obtained for the whole genome in a previous study of this population (Lehtovaara *et al.* 2013) supports a true difference. It is also noteworthy that comparisons between genetic correlations require exceptionally high sample sizes, and differences are rarely expected to be supported statistically (Lynch and Walsh 1998; Bonduriansky and Chenoweth 2009). Thus, our findings suggest that sex-specific modifiers of genes influencing lifespan are overrepresented on the X chromosome. A lower *r*_MF_ at the X chromosome in *Drosophila* has previously been found for cuticular hydrocarbons (Chenoweth and Blows 2003; Chenoweth *et al.* 2008), most likely for some, but not all, of a range of morphological traits (Cowley *et al.* 1986; Cowley and Atchley 1988), and to a small degree for gene expression (Griffin *et al.* 2013).

In the previous study of this population (Lehtovaara *et al.* 2013), the *r*_MF_ for aging was estimated to be close to zero (−0.11 and 0.10 in two social environments) across the entire genome. Therefore, it is unlikely that there is potential for the X chromosome and autosomes to show intersexual genetic correlations departing far from zero. In line with this, we estimate the *r*_MF_ for both the X chromosome and the autosomes to not differ from zero for aging.

### On the potential for the X and the autosomes to contribute to adaptive evolutionary change

Theory predicts that hemizygosity of the X chromosome should result in relatively faster adaptive change from novel beneficial mutations at the X chromosome compared to the autosomes, whenever mutations are at least partly recessive (Charlesworth *et al.* 1987; Connallon *et al.* 2012; Meisel and Connallon 2013; Orr and Betancourt 2001). With respect to adaptive evolution from standing genetic variation, the evolutionary rate is predicted to follow the opposite pattern (Orr and Betancourt 2001). Current evidence favors more rapid evolutionary change on the X chromosome (Meisel and Connallon 2013), but is unable to discern if this results from novel mutations or standing genetic variation.

Our results, for lifespan and aging, suggest that additive genetic variation (if anything) is depleted on the X chromosome (as measured through females). This supports the idea that faster X evolution should result from faster incorporation of X-linked novel mutations rather than from standing genetic variation. The rate of adaptation is, however, dependent on genetic correlations (Lande 1980; Agrawal and Stinchcombe 2009). Positive genetic correlations between the sexes can enhance the response to selection when the sexes are selected concordantly, but have the opposite effect when selection is sexually antagonistic. Similarly, a low genetic correlation impedes the rate of adaptation of traits selected concordantly in the sexes, while it allows for more rapid evolution of sexual dimorphism for traits subjected to sexually antagonistic selection (Lande 1980; Bonduriansky and Rowe 2005; Bonduriansky and Chenoweth 2009; Poissant *et al.* 2010; Lewis *et al.* 2011; Gosden *et al.* 2012; Griffin *et al.* 2013; Ingleby *et al.* 2014). In this respect, our finding of a lower *r*_MF_ for the X chromosome than the autosomes for lifespan suggests that adaptive evolution from standing genetic variation would proceed relatively faster on the X chromosome when driven by sexually antagonistic selection, while proceeding relatively slower when driven by sexually concordant selection. This opens up the possibility that the faster X observed in many studies results from sex-specific selection on standing genetic variation in traits with a low *r*_MF_, a conclusion that fits with the strongest evidence for a faster X effect, which has been observed for genetic factors with sex-biased expression (reviewed in Meisel and Connallon 2013).

## Supplementary Material

Supplemental Material
