# Supplementary material for: Autosomal and X-Linked Additive Genetic Variation for Lifespan and Aging: Comparisons Within and Between the Sexes in Drosophila melanogaster
Source: G3 (Bethesda). 2016 Sep 27;6(12):3903–11. doi: 10.1534/g3.116.028308 (PMC5144961; doi:10.1534/g3.116.028308)
Supplement: Supplemental Material [file supp_6_12_3903__index.html]

Autosomal and X-Linked Additive Genetic Variation for Lifespan and Aging: Comparisons Within and Between the Sexes in Drosophila melanogaster — Supplemental Material 

# Autosomal and X-Linked Additive Genetic Variation for Lifespan and Aging: Comparisons Within and Between the Sexes in *Drosophila melanogaster*

## Supplemental Material for Griffin, *et al*, 2016

**Files in this Data Supplement:**

- Figure S1 - Crosses used to produce chromosome substitution lines. (.pdf, 262 KB)
- Figure S2 - Distribution of mean lifespan across vials for X- and A-lines in males and females. (.pdf, 214 KB)
- Figure S3 - Posterior distributions from MCMCglmm models of lifespan and aging. (.pdf, 232 KB)
- Figure S4 - Predicted total and additive X linked genetic variance in males and females. (.pdf, 218 KB)
- Table S1 - Mean and variance estimates for lifespan and aging including outliers (MCMCglmm). (.pdf, 341 KB)
- Table S2 - Mean and variance for lifespan as estimated by a REML model fit with outliers vials omitted. (.pdf, 246 KB)
