# Supplementary figures and images for: Autosomal and X-Linked Additive Genetic Variation for Lifespan and Aging: Comparisons Within and Between the Sexes in Drosophila melanogaster
Source: G3 (Bethesda). 2016 Sep 27;6(12):3903–11. doi: 10.1534/g3.116.028308 (PMC5144961; doi:10.1534/g3.116.028308)

Figure S1

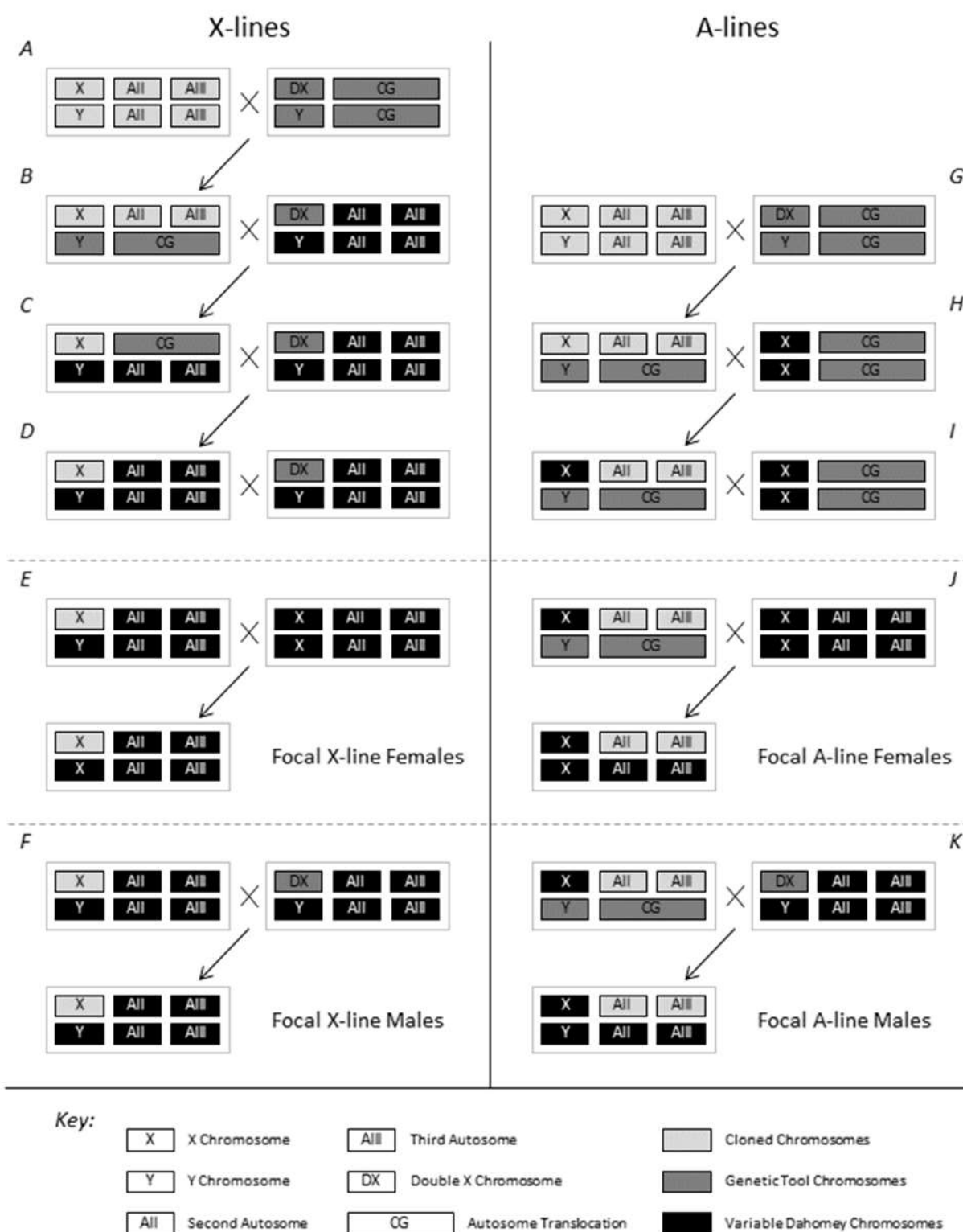

Supplement: Supplemental Material [file supp_g3.116.028308_FigureS1.pdf]

Figure S2

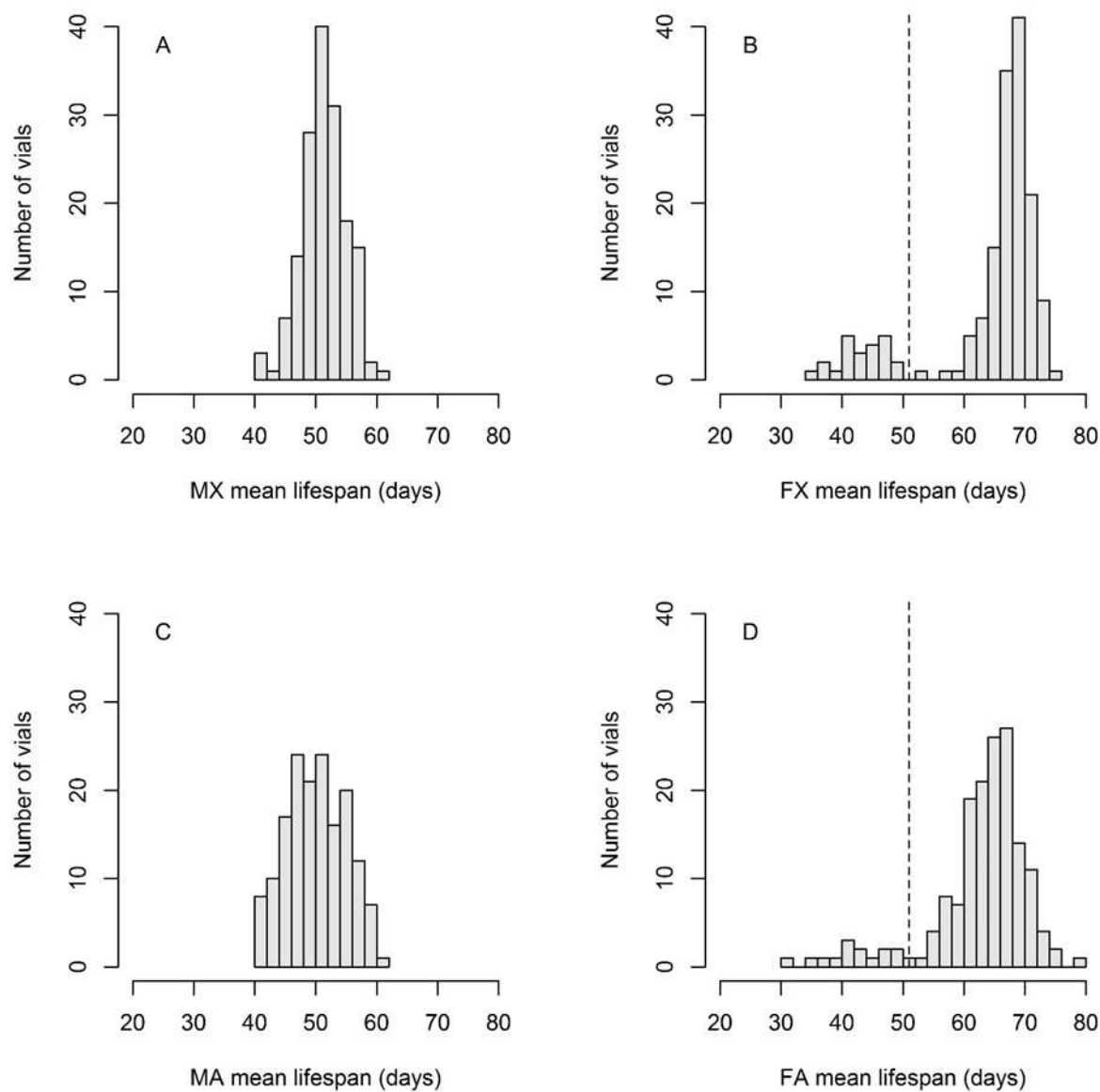

Supplement: Supplemental Material [file supp_g3.116.028308_FigureS2.pdf]

Figure S3

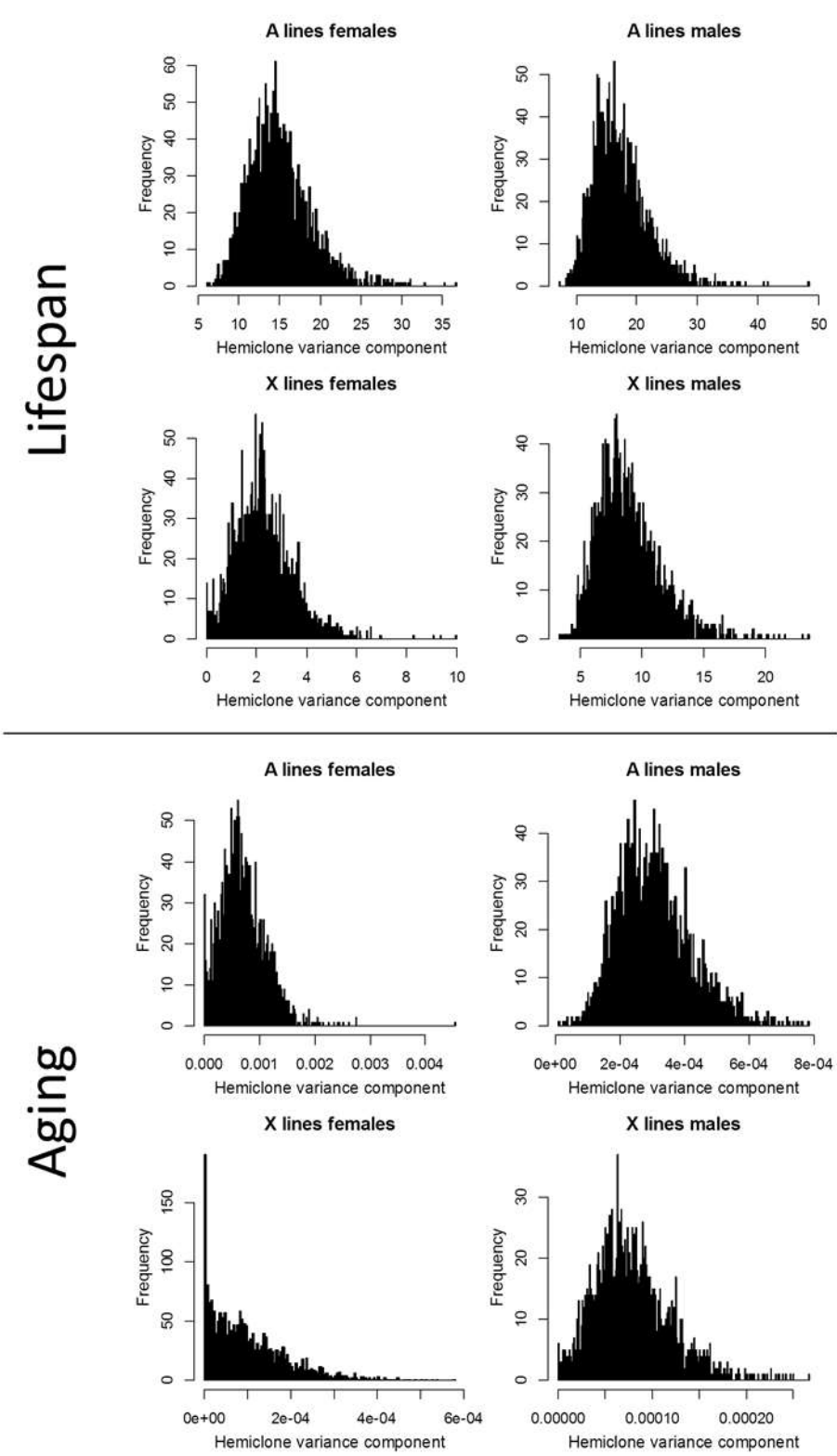

Supplement: Supplemental Material [file supp_g3.116.028308_FigureS3.pdf]

Figure S4

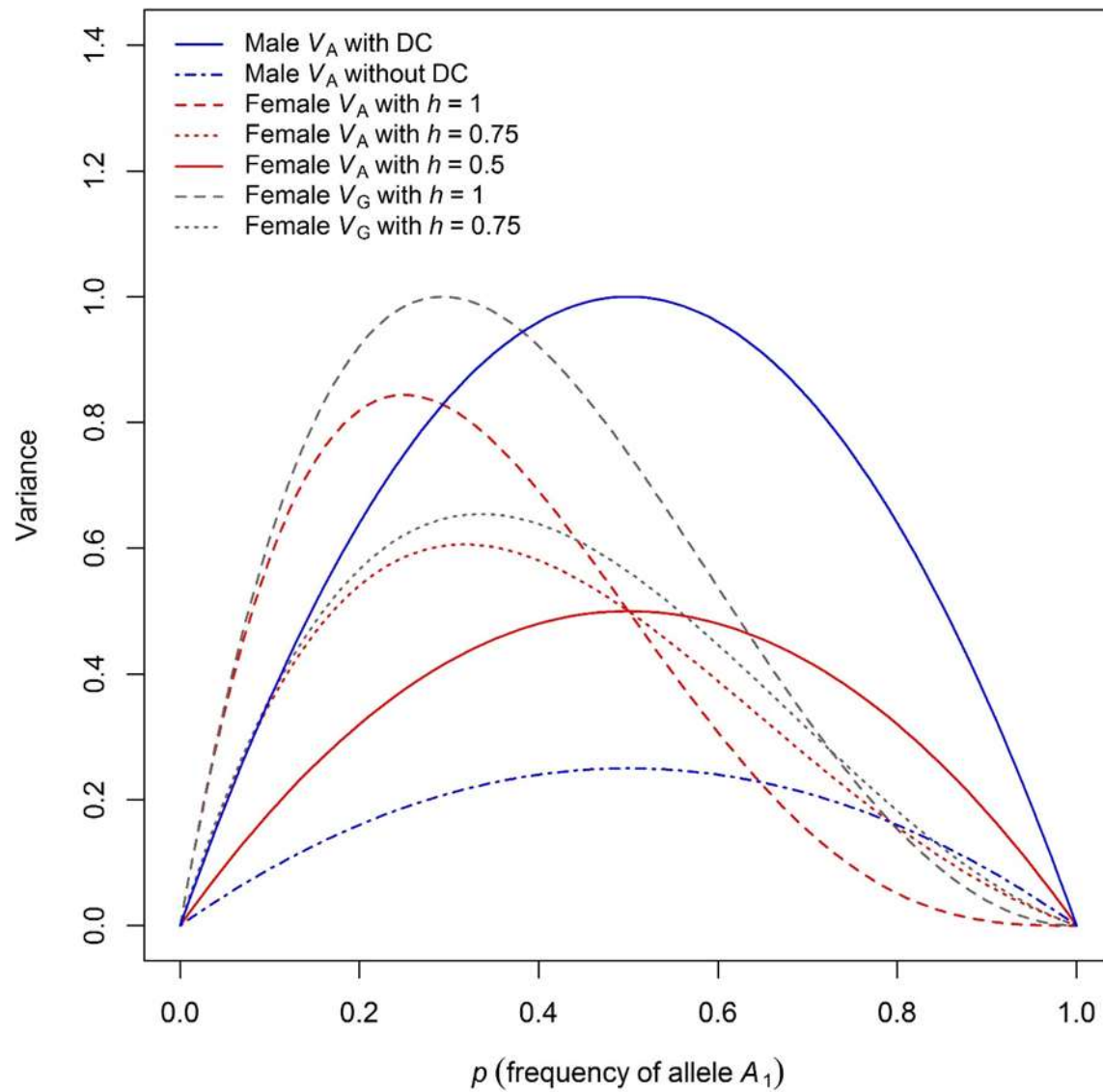

Supplement: Supplemental Material [file supp_g3.116.028308_FigureS4.pdf]
