# Supplementary material for: Autosomal and X-Linked Additive Genetic Variation for Lifespan and Aging: Comparisons Within and Between the Sexes in Drosophila melanogaster
Source: G3 (Bethesda). 2016 Sep 27;6(12):3903–11. doi: 10.1534/g3.116.028308 (PMC5144961; doi:10.1534/g3.116.028308)
Supplement: Supplemental Material [file supp_g3.116.028308_TableS1.pdf]

Table S1. Mean and variance estimates for lifespan and aging including outliers (MCMCglmm). Values provided are the estimates from the model, followed by the standard error, and 95% credible intervals (CI). Mean lifespan is given in days, and mean aging is given for values of β (x 100), with estimates of line (V_L), vial (V_V) (only for lifespan), residual (V_R) and phenotypic (V_P), variance. Additive genetic variance (V_A) and the coefficient of additive genetic (CV_A) were calculated from the line variance and the mean.

		**Autosomes**		**X-chromosomes**	
		**Female**	**Male**	**Female**	**Male**
**Lifespan**	**Mean**	62.72 ± 0.84	50.05 ± 0.7	64.29 ± 0.86	51.38 ± 0.51
	CI	61.1 - 64.38	48.66 - 51.47	62.55 - 65.94	50.39 - 52.4
	V_L	16.61 ± 7.20	17.24 ± 4.60	9.48 ± 7.46	8.93 ± 2.58
	CI	5.47 - 32.97	10.37 - 28.04	0.07 - 27.25	5.08 - 14.71
	V_V	45.58 ± 6.51	4.83 ± 0.88	75.69 ± 10.16	3.18 ± 0.69
	CI	34.77 - 60.21	3.29 - 6.76	57.86 - 98.15	2.03 - 4.67
	V_R	73.88 ± 1.20	89.95 ± 1.46	63.79 ± 1.04	98.93 ± 1.58
	CI	71.57 - 76.21	87.15 - 92.76	61.79 - 65.81	95.82 - 102.15
	V_P	136.07 ± 8.46	112.03 ± 4.85	148.96 ± 10.33	111.04 ± 3.06
	CI	121.91 - 155.06	104.46 - 123.08	131.65 - 170.8	106.00 - 117.69
	V_A	33.22 ± 14.41	34.49 ± 9.19	18.96 ± 14.92	8.93 ± 2.58
	CI	10.94 - 65.93	20.74 - 56.08	0.14 - 54.5	5.08 - 14.71
	CV_A	0.090 ± 0.020	0.116 ± 0.015	0.062 ± 0.028	0.058 ± 0.008
	CI	0.053 - 0.130	0.091 - 0.150	0.006 - 0.115	0.044 - 0.075
**Aging (β)**	**Mean**	16.84 ± 0.57	12.39 ± 0.34	14.17 ± 0.32	10.97 ± 0.2
	CI	15.71 - 17.99	11.73 - 13.05	13.52 - 14.83	10.58 - 11.36
	V_L	6.22 ± 3.28	3.07 ± 1.25	0.77 ± 0.79	0.77 ± 0.40
	CI	0.65 - 13.48	1.11 - 6.03	0.00 - 2.76	0.13 - 1.68
	V_R	26.09 ± 3.57	6.68 ± 0.90	13.72 ± 1.65	3.03 ± 0.41
	CI	20.11 - 33.83	5.14 - 8.62	10.80 - 17.15	2.32 - 3.94
	V_P	32.31 ± 3.97	9.76 ± 1.34	14.48 ± 1.67	3.80 ± 0.48
	CI	25.38 - 40.51	7.50 - 12.87	11.51 - 18.20	3.00 - 4.85
	V_A	12.44 ± 6.56	6.14 ± 2.49	1.54 ± 1.57	0.77 ± 0.40
	CI	1.30 - 26.96	2.21 - 12.05	0.01 - 5.52	0.13 - 1.68
	CV_A	0.201 ± 0.060	0.196 ± 0.041	0.076 ± 0.044	0.077 ± 0.021
	CI	0.068 - 0.310	0.119 - 0.279	0.005 - 0.166	0.033 - 0.119
