# Supplementary material for: Autosomal and X-Linked Additive Genetic Variation for Lifespan and Aging: Comparisons Within and Between the Sexes in Drosophila melanogaster
Source: G3 (Bethesda). 2016 Sep 27;6(12):3903–11. doi: 10.1534/g3.116.028308 (PMC5144961; doi:10.1534/g3.116.028308)
Supplement: Supplemental Material [file supp_g3.116.028308_TableS2.pdf]

Table S2. Mean and variance for lifespan as estimated by a REML model fit with outliers vials omitted. Values provided are the point estimates and standard errors from the multivariate model fitted in ASReml for lifespan and univariate models fitted in R (package lme4, SE estimated by parametric bootstrapping) for aging.

		Autosomes		X-chromosomes	
		**Female**	**Male**	**Female**	**Male**
**Lifespan**	**Mean**	64.78	49.99	67.79	51.36
	V_L	13.79 ± 3.52	15.95 ± 3.99	2.18 ± 0.97	8.03 ± 2.11
	V_V	4.64 ± 0.86	4.65 ± 0.87	5.55 ± 0.98	3.06 ± 0.66
	V_R	73.72 ± 1.24	89.94 ± 1.46	60.94 ± 1.05	98.86 ± 1.58
	V_P	92.15 ± 3.77	110.54 ± 4.28	68.67 ± 1.56	109.95 ± 2.66
	V_A	27.58 ± 7.05	31.9 ± 7.97	4.35 ± 1.95	8.03 ± 2.11
**Aging**	**Mean**	17.27	12.41	14.76	10.96
	V_L	6.71 ± 3.27	2.83 ± 1.01	0.62 ± 0.81	0.76 ± 0.35
	V_R	24.36 ± 3.45	6.34 ± 0.85	12.43 ± 1.71	2.79 ± 0.36
	V_P	31.07 ± 3.79	9.16 ± 1.22	13.04 ± 1.68	3.54 ± 0.42
	V_A	13.42 ± 6.54	5.65 ± 2.02	1.23 ± 1.63	1.51 ± 0.70
